# Genetic background of behavior traits in lactating sows under heat-stress conditions and their relationship with heat tolerance and maternal performance traits

**DOI:** 10.3389/fgene.2025.1688262

**Published:** 2025-11-17

**Authors:** Sharlene O. Hartman, Lorena F. Benfica, Jay S. Johnson, Jeremy N. Marchant, Hinayah R. Oliveira, Victor B. Pedrosa, Allan P. Schinckel, Yijian Huang, Leonardo S. Gloria, Hui Wen, Luiz F. Brito

**Affiliations:** 1 Department of Animal Sciences, Purdue University, West Lafayette, IN, United States; 2 Division of Animal Science, University of Missouri, Columbia, MO, United States; 3 Organic Plus Trust, Alexandria, VA, United States; 4 A World of Good Initiative Inc., Dover, DE, United States; 5 Smithfield Premium Genetics, Rose Hill, NC, United States

**Keywords:** behavioral genomics, genetic correlation, heat stress, heritability, maternal-line pigs

## Abstract

Heat stress is among the most significant welfare challenges facing modern swine production systems worldwide. Pigs are particularly susceptible to heat stress due to their inactive sudoriferous glands, which limits their capacity for evaporative cooling. As a result, they rely predominantly on behavioral strategies for thermoregulation. This behavioral dependence underscores the potential value of integrating behavioral assessments with genetic analyses to identify heritable components of climatic resilience. In this context, the main objectives of this study were as follows: 1) to develop an ethogram to evaluate the response of lactating sows to a novel event (i.e., hair shaving); 2) to derive the traits’ responsiveness score (RS), vocalization score (VS), and shave time (ST) from the ethogram, and identify key systematic effects influencing these behavioral responses of lactating sows under heat-stress conditions; 3) to estimate variance components for all the derived traits; 4) to assess genetic correlations between the behavioral traits and both direct indicators of heat tolerance and maternal ability traits; and 5) to perform genome-wide association studies (GWAS) to identify genomic regions associated with sow behavioral traits. RS, VS, and ST were found to be heritable with heritability estimates of 0.17 ± 0.05, 0.15 ± 0.05, and 0.10 ± 0.05, respectively. These traits had null-to-low genetic correlations with maternal performance and low-to-moderate genetic correlations with direct indicators of heat tolerance. Twelve genomic markers were found to be significantly associated with the three behavioral traits, including regions overlapping with genes known to influence heat stress response, such as *PIK3R5* and *NCF2*. In conclusion, sow behavioral responses to a novel event under heat-stress conditions are heritable and highly polygenic but uncorrelated or lowly correlated with climatic resilience and maternal traits.

## 1 Introduction

An increase in global temperatures poses widespread welfare and productivity challenges for livestock (Baumgard and Rhoads, 2013; Rojas-Downing et al., 2017). Domestic pigs are especially sensitive to heat stress due to their evolutionary loss of functional sudoriferous glands and intense genetic selection for growth and efficiency traits (Ramirez et al., 2022), with high temperatures adversely affecting their production, health, and overall well-being (Johnson, 2018). Behavior is a fundamental component of animal welfare assessment. Compared to physiological indicators, behavioral observations are often less invasive and provide direct insights into an animal’s needs, preferences, and interactions with its environment (Tuyttens et al., 2014; Manteuffel et al., 2021). For instance, Huynh et al. (2005) observed that pigs exposed to hotter and more humid environments spent more time lying down, whereas Cronin et al. (1996) demonstrated that postural changes in sows were associated with farrowing success and maternal responsiveness, including lower piglet crushing and greater movement in response to distress calls. To quantify such behavioral responses, most studies have used ethograms, which are structured catalogs of behaviors tailored to the species and environmental context (Bateson and Martin, 2021). For ethograms to be effective, they must include sufficient behavioral detail to capture meaningful variation (McGlone, 1986; Jensen, 1980), as demonstrated in prior studies linking specific behavioral patterns with physiological stress indicators (e.g., Poletto et al., 2010; Pepin et al., 2021).

Most studies investigating the genetic background of heat tolerance in pigs were based on routinely recorded performance traits and climatic data from public weather stations (Freitas et al., 2021; Tiezzi et al., 2020) or physiological and anatomical indicators of heat stress response (Johnson et al., 2023; Freitas et al., 2023; Wen et al., 2023; 2024). However, there is a need for investigating individual sow behavioral response and their genetic links with thermotolerance. Ethograms and behavioral assessment protocols can contribute to the identification of auxiliary traits for optimizing genetic selection for improved heat tolerance. More balanced selection indices for commercial swine herds represent a key strategy to enhance animal welfare by breeding pigs that are more resilient to environmental challenges (Hermesch et al., 2015; Berghof et al., 2019). However, the current understanding of the biological mechanisms and genetic factors underlying heat stress responses in lactating sows remains limited. In this context, the main objectives of this study were as follows: 1) to develop an ethogram to assess lactating sow behavioral response during hair shaving under commercial farm conditions; 2) to derive behavioral traits based on an ethogram and identify the most significant systematic effects influencing sow behavioral responses under heat-stress conditions; 3) to estimate variance components for all the behavioral traits in a maternal-line (Landrace × Large White) pig population; 4) to assess the genetic relationship of the behavioral traits with direct indicators of heat tolerance, including vaginal and skin temperature, respiration rate, and panting score, along with indicators of reproduction and maternal ability traits; and 5) to perform genome-wide association studies (GWAS) to identify genomic regions associated with sow behavioral responses during hair shaving. We hypothesized that ethogram-based behavioral response during exposure to novel events (e.g., hair shaving procedures) may be heritable and genetically correlated with sow climatic resilience and reproductive performance. More specifically, we expected that sows displaying stronger behavioral reactivity to the employed test would also show higher thermal load and that such behavioral sensitivity could be genetically associated with reduced maternal performance under heat-stress conditions.

## 2 Materials and methods

The experimental protocol followed ethical principles in animal research (Federation of Animal Science Societies, 2020) and was approved by the Purdue University Animal Care and Use Committee (Protocol #1912001990).

### 2.1 Animals and genotypes

Phenotypes and genotypes were collected on 1,678 multiparous (from parity 2 to 7) lactating sows (Landrace × Large White) housed in barns at a commercial production farm in Maple Hill, North Carolina, United States (34.70738°, −77.73653°), during the months of June and July of 2021, as described by Freitas et al. (2023) and Johnson et al. (2023). In-barn dry bulb temperature and relative humidity were continuously recorded, with averages of 26.38 °C ± 1.21 °C and 83.38% ± 5.40%, respectively. The animals were genotyped using the PorcineSNP50K Bead chip (Illumina Inc., San Diego, CA, United States) containing 50,703 single-nucleotide polymorphisms (SNPs). Quality control (QC) of the genomic data involved the removal of SNPs and individuals with a call rate below 0.90, SNPs with a minor allele frequency (MAF) less than 0.01, SNPs exhibiting a deviation between observed and expected heterozygosity greater than 0.15, and those with missing or duplicated genomic positions. Genotyped animals with no phenotypic records were also discarded. After the QC, 45,028 SNPs for 1,625 animals remained for subsequent analyses.

### 2.2 Phenotypic data

Behavior and heat stress response traits were collected in lactating sows (parities 2 through 7) between 6 and 19 days of lactation, in addition to maternal ability traits. The traits evaluated included responsiveness score (RS), shave time (ST), vocalization score (VS), total number of piglets born (TB), number of piglets born alive (LB), number of stillborn piglets (SB), number of piglets weaned (PW), number of piglet deaths (PD), vaginal temperature (VT), respiration rate (RR), and panting score (PS).

The sows and their piglets were housed in a commercial facility in standard farrowing crates (2.0 × 1.8 m) within a farrowing pen. Each farrowing crate was equipped with a feeder, an individual auger spout, and a nipple waterer. Piglets had continuous access to their dam and could enter the area enclosed by the crate for nursing or exit to the outer section, which was equipped with electric heating pads. All sows included in this study were shaved along their sides to enable accurate skin temperature measurements. This procedure constituted a standardized instance of human-mediated handling that was consistently applied across all animals. A detailed ethogram was developed (Tables 1, 2, 3) to systematically capture behavioral responses exhibited during the shaving sessions through direct observation by trained individuals. Shaving was performed weekly (on different animals) over a 6-week period, with procedures beginning no earlier than 0800 h and concluding no later than 1500 h. Each week, between 202 and 282 sows were shaved on the same day to maintain procedural consistency. Although interobserver reliability was not formally tested, all observers participated in an intensive joint training using pilot animals until full consensus on scoring criteria was achieved, following the recommendations of Tuyttens et al. (2014). Observers recorded behaviors simultaneously under the same environmental and procedural conditions, minimizing subjective variation.

**TABLE 1:** Ethogram for assessing the behavioral responses to shaving in lactating sows under heat-stress conditions.

Behavior	Definition	Abbreviation
Posture		
Laying down laterally	Laying on a side with one shoulder in contact with the ground. Legs horizontally stretched. Belly line visible. Side of the head in contact with the ground	LDL
Laying down sternally	Laying with the sternum in contact with the ground. Legs are/are mostly tucked in. Belly line is not/partially visible. Side of the head is not in contact with the ground	LDS
Sitting	Front legs straight and front feet in contact with the ground. Hind legs and rump in contact with the ground	SIT
Standing	All four hooves on the ground. Body in the upright position	STD
Vocalizations		
Short grunt	Low vocalizations produced with the respiration rate of the animal	SGT
Long grunt	Drawn vocalization that interrupts animal’s previous respiration rate	LGT
Bark	Loud short vocalization resembling the sudden bark of a dog	BRK

**TABLE 2:** Scoring system used for assessing postural changes observed in lactating sows under heat-stress conditions.

Score	Posture change^a^
−3	From STD to LDL
−2	From STD to LDS or SIT to LDL
−1	From STD to SIT or SIT to LDS or LDS to LDL
0	No postural changes
1	From LDL to LDS or LDS to SIT or SIT to STD
2	From LDL to SIT or LDS to STD
3	From LDL to STD

^a^ All abbreviations are described in Table 1.

**TABLE 3:** Scoring system used for assessing vocalization types observed in lactating sows under heat-stress conditions.

Score	Vocalization behavior^a^
0	No vocalization
1	SGT only
2	SGT and LGT
3	LGT only
4	SGT, LGT, and BRK
5	SGT and BRK
6	LGT and BRK
7	BRK only

^a^ SGT, short grunt; LGT, long grunt; BRK, bark.

Five research workers were trained to perform the shaving procedure according to a standardized protocol. Using electric hair clippers, they removed a hand-sized area of hair from both sides of the rump while positioned behind the animal and outside the farrowing crate. Hair shaving began on the most accessible side, and if necessary, gentle physical prompts were used to encourage the sow to reposition to access the opposite side. Four trained behavior observers were stationed in front of each animal and recorded its posture at three key time points: (1) prior to any physical contact, when the sow was surrounded by the research workers, (2) following the completion of shaving on the first side, and (3) after the second side had been shaved completely. After a sow was shaved, the recorders and shavers proceeded to the next animal, continuing the procedure sequentially across rooms containing up to four rows, each row with a capacity of up to 14 sows. However, not all animals within each room were included in the study as differences in farrowing dates resulted in some sows falling outside the targeted lactation window for data collection (6–19 days of lactation).

Behavioral traits were measured during the shaving process. Behaviors were later scored based on postural changes and vocalization patterns. Postures were scored according to the sow’s postural changes throughout shaving. The postures were classified as lying down laterally (LDL), lying down sternally (LDS), sitting (SIT), or standing (STD) at each of these stages, and can be described as ranging in a hierarchical fashion from least alert (LDL) to most alert (STD). As the animal’s posture was recorded at three distinct phases, the posture score change was determined by comparing the current posture with the previous posture and assigning a numerical value based on the direction and magnitude of the change. These values ranged from −3 to 3, with the four positions treated as categorical levels, as described in Tables 1, 2. For example, if a sow changed posture from LDS to STD, it would be a change in the more alert direction and by two postural categories, so the score would be 2. A change from STD to LDS would be a change in the less alert direction and again by two postural categories, so the score would be −2.

The RS was determined by combining the VS and posture score. If an animal increased its alertness by sitting or standing up from its original position, it received a positive score, contributing to a higher RS. Conversely, if the animal sat or laid down during the handling test, it received a negative score. Sows that remained in the same position were assigned a score of 0, indicating a less reactive animal. ST was measured in seconds and refers to the total time taken to completely shave both sides of each sow. Recorders also annotated the types of vocalizations produced by the sows across four stages of the shaving process: 1) initially, before the shavers touched the animal and when they were positioned in front and behind the sow, 2) during the shaving of the first site, 3) when the animal was encouraged to change the position (if necessary), and 4) during the shaving of the second site. Vocalizations were initially categorized into four types: no vocalization, short grunt (SGT), long grunt (LGT), and bark (BRK). Detailed definitions of these behaviors are provided in the ethogram presented in Table 2. Shavers scored vocalizations at three or four stages of the shaving process, and animals received respective scores at each stage. These individual scores were later summed up to form VS, as shown in Table 3. This scale also reflects a hierarchical gradient of alertness, ranging from no vocalization (indicating the lowest level of alertness), progressing through short grunts and long grunts, to barks, which represent the highest level of alertness.

The sow maternal traits included in this study were as follows: LB, SB, PW, PD, and TB, which is the sum of LB and SB. The records for these traits were obtained from farm records and pertained to the litter and lactation associated with each sow during their participation in the study. Cross-fostering records were not available in the dataset used. Additional traits included in this study were automatically recorded: VT, RR, and PS, as described by Johnson et al. (2023) and Freitas et al. (2023).

Animal records were discarded if the sows were not fully shaved due to shavers being unable to access both sides of their body. Outliers of continuous scale traits were removed if they had values greater than three standard deviations from the mean (n = 4). Outliers in categorically recorded traits were evaluated for the biological plausibility of their observed values, but no records were removed. A total of 1,297 sows had complete behavioral records, whereas 1,593 sows had complete maternal performance records through weaning, and 1,609 sows had complete records through farrowing.

### 2.3 Statistical analyses

The systematic effects considered in the statistical model for each trait were determined through a stepwise model selection procedure based on backward and forward elimination (significance threshold P < 0.05). Full models were initially fitted using the lm() function in R (R Core Team, 2020), and model refinement was subsequently performed with the stepAIC() function from the MASS package (Venables and Ripley, 2002), allowing both backward and forward selection to iteratively remove or add nonsignificant effects and identify the most parsimonious model. The effects considered for each trait are described in Table 4. All significant systematic effects were included in the models used for variance component estimation with the BLUPf90 family of programs (Misztal et al., 2014). Heritability estimates were calculated based on single-trait animal model analyses, and genetic and phenotypic correlations between all trait pairs were calculated based on two-trait analyses. Traits with repeated records (i.e., VT, RR, and PS) were analyzed using a repeatability linear animal model, and single-record traits were analyzed using a linear animal model. The analyses were performed using GIBBSf90+ software from the BLUPF90 family of programs (Misztal et al., 2014). A chain containing 500,000 iterations, with burn-in of 150,000 and thinning interval of 80 iterations, was used for all trait combinations. The model convergence for all analyses was assessed based on visual inspection of the trace plots of each component and Geweke convergence criteria (p-value exceeding 0.05) using the “boa” R package (Smith, 2007). Effective sample sizes were calculated for all parameters, with an average effective size of 4,375 across traits, which was considered sufficient to ensure reliable posterior estimates.

**TABLE 4:** Systematic effects included in the final statistical models fitted for each trait.

Trait^a^	Systematic effect(s)^b^	Covariate(s)^c^	Random effect(s)^d^
RS	CG, IPT, FPT, SPT, IM, and HD	TT	a
VS	CG, IPT, FPT, SPT, IM, and HD	TT	a
ST	CG, FPT, Par, and HD	WS-T and TT	a
LB	Par and FCG		a
TB	Par and FCG		a
SB	Par and FCG		a
PW	Par and WCG		a
PD	Par and WCG		a
VT	Par, Wk, and loc	DP	a and pe
RR	RRR, Wk/D/T, Par, and loc	DIL and WS-T	a and pe
PS	PSR, Wk/D, Par, and loc	DIL and WS-T	a and pe

^a^ RS, responsiveness score; ST, shave time; VS, vocalization score; LB, live born; TB, total born; SB, still born; PW, pigs weaned; PD, pig deaths; VT, vaginal temperature; RR, respiration rate; PS, panting score.

^b^ CG, contemporary group; IPT, initial position type; FPT, first shave site position type; SPT, second shave site position type; IM, intervention moment; HD, hair density score; Par, parity; FCG, farrowing contemporary group; WCG, weaning contemporary group; Wk, week; loc, location of the sow based on the building and room within that the animal was housed; Wk/D/T, a concatenation of the week, day, and the time in hour of the day; RRR, respiration rate recorder; PSR, panting score recorder; Wk/D, a concatenation of the week with the day.

^c^ DIL, days in lactation; WS-T, weather station temperature; DP, dew point; TT, total touches applied by the ethogram shaver.

^d^ a, animal genetic effect; pe, permanent environmental effect.

The statistical models for single (Equation 1) and repeated (Equation 2) record traits can be described as follows:

y=Xβ+Za+e, (1)

y=Xβ+Za+Wpe+e, (2)

where y is the vector of phenotypic records, β is the vector of systematic effects specific to each trait, and a is the vector of random animal additive genetic effects, assumed as a ∼ N (0, Gσ^2^_a_), where σ^2^_a_ is the additive genetic variance for each trait. The genomic relationship matrix (G) was constructed using the first method proposed by VanRaden (2008). e is the vector of residual effects assumed as e ∼ N (0, Iσ^2^_e_), where σ^2^_e_ is the residual variance; pe is the vector of random permanent environmental effects, defined as pe ∼ N (0, Iσ^2^_pe_), where σ^2^_pe_ is the permanent environmental variance; and X, Z, and W are the incidence matrices linking the phenotypic records to the systematic, direct additive genetic, and permanent environmental effects, respectively.

**FIGURE 1:**
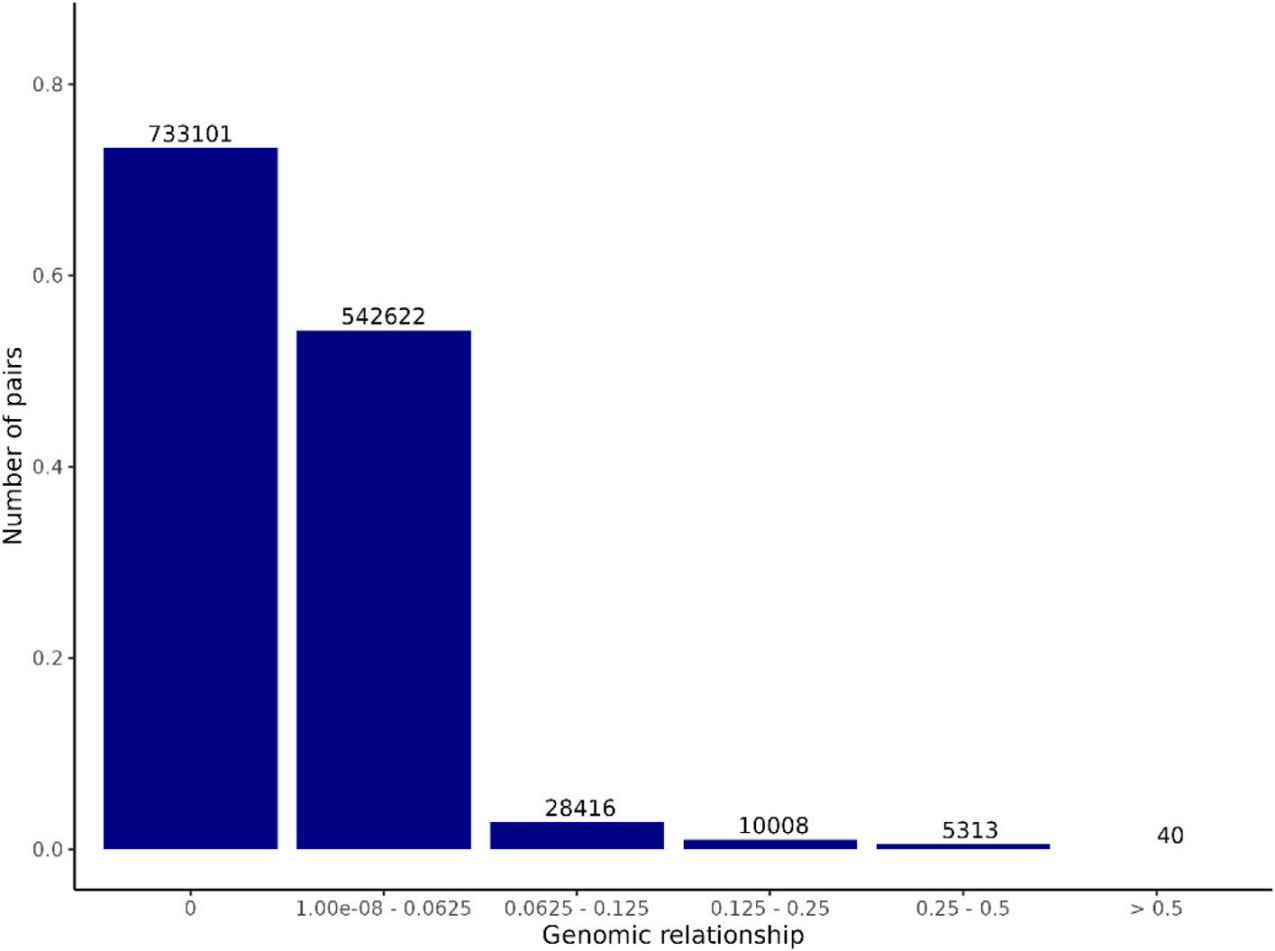
Distribution of pairwise genomic relationship coefficients in the studied population. Values were obtained from the upper triangle of the genomic relationship matrix (without considering the diagonal elements).

As pedigree information was not recorded in this population, we assessed population structure parameters based on G (pairwise relationships among animals). In particular, values from the upper triangle (excluding the diagonal) of the G matrix were extracted. The distribution of these coefficients is shown in Figure 1, illustrating the degree of relatedness and the overall genetic diversity within the population.

The heritability (h^2^) estimates for the single (Equation 3) and repeated (Equation 4) record traits were calculated as follows:

$$h^{2}=\frac{\sigma_{a}^{2}}{\sigma_{a}^{2}+\sigma_{e}^{2}},$$ (3)

$$h^{2}=\frac{\sigma_{a}^{2}}{\sigma_{a}^{2}+\sigma_{\text{pe}}^{2}+\sigma_{e}^{2}},$$ (4)

where $\sigma_{a}^{2}$ , $\sigma_{pe}^{2}$ , and $\sigma_{e}^{2}$ were previously defined. The genetic correlations (r_g_) between each pair of traits were calculated as follows: $r_{g}=\frac{{COV}_{12}}{\sqrt{\sigma_{a1}^{2}\;*\;\sigma_{a2}^{2}}}$ , where COV_12_ is the genetic covariance between trait 1 and trait 2, $\sigma_{a1}^{2}$ is the additive genetic variance for trait 1, and $\sigma_{a2}^{2}$ is the additive genetic variance for trait 2.

### 2.4 Genome-wide association studies and functional analyses

Genome-wide association studies were performed for RS, VS, and ST, using postGSf90 software (Aguilar et al., 2014). Initially, variance components were estimated and genomic estimated breeding values (GEBVs) were predicted. Subsequently, the postGSf90 package was used to back-solve GEBVs into SNP effects and estimate approximate p-values. Chromosome-wise significance thresholds for the GWAS’ results were determined based on a Bonferroni correction at α = 0.05 and considering the number of independent chromosomal segments (Me) to account for the dependence among tests due to LD. Me was calculated as proposed by Goddard et al. (2011):

$$\text{Me}=\frac{2\text{NeL}}{\log\left(\text{NeL}\right)},$$

where Ne is the effective population size (Ne = 100) and L is the length of each chromosome expressed in Morgans. An SNP was considered statistically significant if its p-value was smaller than 0.05/Me, which ranged from 8 × 10^−6^ to 7.79 × 10^−4^. Furthermore, the genomic inflation factor (λ) was calculated to quantify any deviation from the expected p-value distribution, potentially due to biases such as inadequate control of population structure effects. The final genomic inflation factor was 1.01 for the three traits, indicating minimal genomic inflation (Supplementary Material 1).

Following the GWAS, the GALLO R package (Fonseca et al., 2020) was used for gene annotation, and the “gprofiler” R package was used for functional enrichment analyses (Kolberg et al., 2020). Gene annotation and enrichment processes were performed using a genomic window of 100 kb upstream and downstream of the most significant identified SNPs, based on the ensemble database for *Sus scrofa* (Martin et al., 2023). The Quantitative Trait Loci identification and functional enrichment was based on the Pig QTL database from the Animal QTL Database (Hu et al., 2022). All significance thresholds were set to a p-value of 0.05 or lower.

## 3 Results

Table 4 summarizes the systematic effects included in the models. For behavioral traits, the fixed effects considered were as follows: contemporary group, position during shaving, moment of intervention, and number of touches. For maternal traits, only contemporary group and parity were included. For heat stress response traits, the model included recorder, day and week of measurement, time of measurement, days in lactation, and location. Parity was included as a fixed effect across all trait groups, whereas ambient temperature was included as a covariate for both behavioral and heat stress traits.

Table 5 shows the descriptive statistics for the traits evaluated in this study. The coefficients of variation for RS, VS, and ST were 58%, 66%, and 48%, respectively. The RS across all animals ranged from 0 to 15, with ∼18% of the sows having a score of 3, and an overall mean of 5.73. For VS, 26% of animals had a score of 3, with a range of 0–20 and an overall average of 4.53. ST ranged from 12 s to 3 min and 37 s, with nearly 55% of animals taking between 29 and 59 s to be shaved. The average time taken to shave a sow was 55.32 s. Of the 1,297 animals shaved under the standardized handling procedure, approximately 44% had initial positions that allowed direct access to both shaving sites or adjusted their position in response to handling, eliminating the need for intervention. Among the 724 animals that required manual repositioning by the shaver, 17.7% initially had both sites inaccessible and, therefore, experienced intervention before the shaving of the first site. The remaining 82.3% had one site accessible but required intervention to access the second site.

**TABLE 5:** Descriptive statistics of behavior and maternal traits recorded in lactating sows under heat-stress conditions.

Trait	Min	Median	Mean	Max	SD	Animals with records
Responsiveness score	0	5	5.74	15	3.35	1,297
Vocalization score	0	4	4.54	20	2.99	1,297
Shave time (sec.)	12	50	55.32	217	26.65	1,297
Number of piglets born alive	0	14	13.6	23	3.19	1,609
Total number of piglets born	5	15	14.93	24	3.17	1,609
Number of stillborn piglets	0	0	0.83	11	1.17	1,609
Number of piglets weaned	1	11	11.12	16	2.30	1,593
Number of piglet deaths	0	2	2.62	14	2.43	1,593

Min, minimum value; Max, maximum value; SD, standard deviation; sec, seconds.

Animals were categorized into three groups based on the use and timing of intervention during the shave handling procedure. Among the 573 animals that did not require intervention to change their position, the majority (41%) received a VS of 3, whereas 33.5% scored between 0 and 2, and the remaining animals scored between 4 and 14. A total of 596 animals required intervention between the shaving of the two sites; among them, the majority (17.4%) scored 4, 30.4% scored below this value, and 52.2% scored above 4, with the highest recorded score in this group being 17. Last, 128 animals received intervention before shaving either site; within this group, 19.5% of animals scored 5, 11.7% scored between 2 and 4, and 68.8% scored above 5, with a maximum score of 20 observed.

The hair-shaving procedure was chosen as a controlled and repeatable human-mediated event to elicit a mild behavioral response under heat-stress conditions. Shaving is commonly used on swine farms to facilitate thermographic and skin-temperature assessments and, therefore, represents a practical proxy for evaluating behavioral reactivity to mild discomfort during thermal load. Because all sows were exposed to the same standardized stimulus and handled by trained personnel, differences in responsiveness, posture, and vocalization were interpreted as indicators of coping ability rather than general temperament. Moreover, by combining these behavioral scores with physiological indicators of heat tolerance (vaginal temperature, respiration rate, and panting score), we aimed to capture multidimensional aspects of resilience to heat stress rather than simple aversion to human presence. Future studies should also evaluate additional behavioral tests that may better capture individual behavioral responses more directly related to heat stress responses.

For the maternal traits, the average (±SD) LB was 13.6 ± 3.19, whereas the average TB was 14.93 ± 3.17. The mean SB was 0.83 ± 1.17, and the average PW was 11.12 ± 2.30. On average, 2.62 ± 2.43 piglets per litter died prior to weaning. The sows included in the study were from parities 2 to 7, with approximately 23% of the animals in their second parity, 18% in their third parity, and the remaining 59% in their fourth or later parities.

Table 6 shows the estimates of variance components and heritability for all studied traits. Results were categorized using the following heritability scale: low: from 0.01 to 0.14; moderate: from 0.15 to 0.39; and high: greater than or equal to 0.40 (Wilson et al., 2020). Overall, all traits were found to have low-to-moderate heritability, with values ranging from 0.05 (PS) to 0.17 (RS). The behavior traits had the highest heritability values, with 0.17, 0.15, and 0.10 for RS, VS, and ST, respectively. Table 7 shows the phenotypic (above the diagonal) and genetic (below the diagonal) correlations among the 11 traits analyzed in this study. The highest genetic correlation (0.99 ± 0.01) was observed between RS and VS. The behavior traits did not exhibit significant genetic correlations with maternal traits. However, RS and VS had moderate positive correlations with RR and PS, ranging from 0.33 (RS × PS) to 0.41 (VS × RR). ST was the only trait with a moderate genetic correlation with VT, with an estimated value of 0.52 (0.26). Overall, maternal traits were genetically correlated among themselves, with estimates ranging from −0.64 between LB and SB to 0.97 between LB and TB. PW was the only maternal trait that did not exhibit significant genetic correlations with the other maternal traits. Additionally, maternal traits showed negative genetic correlations with RR and PS, with the strongest correlations observed between TB and RR (−0.62 ± 0.32), between PW and PS (−0.62 ± 0.24), and between RR and LB (−0.44 ± 0.44). Regarding phenotypic correlations, the highest observed value was 0.98 ± 0.00 between RS and VS. Maternal traits exhibited phenotypic correlations ranging from −0.10 (SB and PW) to 0.83 (LB and TB). The remaining phenotypic correlations were close to zero.

**TABLE 6:** Variance components and heritability estimates for sow behavior, reproductive performance, and heat stress response traits.

Trait^a^	$\sigma_{a}^{2}\pm \text{SD}$	$\sigma_{e}^{2}\pm \text{SD}$	$\sigma_{p}^{2}\pm \text{SD}$	$\sigma_{\text{pe}}^{2}\pm \text{SE}$	$h^{2}\pm \text{PSD}$
RS	0.75 ± 0.21	3.62 ± 0.22	4.38 ± 0.18	NA	0.17 ± 0.05
VS	0.76 ± 0.24	4.18. ± 0.25	4.94 ± 0.21	NA	0.15 ± 0.05
ST	20.35 ± 9.37	174.66 ± 10.66	195.00 ± 8.01	NA	0.10 ± 0.05
LB	0.53 ± 0.28	9.21 ± 0.41	9.73 ± 0.35	NA	0.05 ± 0.03
TB	1.10 ± 0.36	8.92 ± 0.43	10.02 ± 0.36	NA	0.11 ± 0.04
SB	0.11 ± 0.04	1.20 ± 0.06	1.31 ± 0.05	NA	0.08 ± 0.03
PW	0.44 ± 0.18	4.54 ± 0.22	4.97 ± 0.18	NA	0.09 ± 0.04
PD	0.66 ± 0.19	4.89 ± 0.23	5.55 ± 0.20	NA	0.12 ± 0.03
VT	0.06 ± 0.01	0.32 ± 0.00	0.51 ± 0.01	0.13 ± 0.01	0.12 ± 0.02^b^
RR	34.64 ± 6.80	443.82 ± 4.45	549.45 ± 6.94	70.99 ± 6.32	0.06 ± 0.01^b^
PS	0.05 ± 0.02	0.84 ± 0.02	1.03 ± 0.02	0.18 ± 0.02	0.05 ± 0.02^b^

^a^ RS, responsiveness score; ST, shave time; VS, vocalization score; LB, total number of piglets born alive; TB, total number of piglets born; SB, total number of stillborn piglets; PW, number of piglets weaned; PD, number of piglet deaths; VT, vaginal temperature; RR, respiration rate; PS, panting score.

^b^ These are estimates and standard errors as reported by Freitas et al. (2023).

**TABLE 7:** Phenotypic correlations shown in the above diagonal and genotypic correlations shown in the below diagonal for all traits and respective posterior standard deviations (PSD) in parenthesis.

	RS (PSD)	VS (PSD)	ST (PSD)	LB (PSD)	TB (PSD)	SB (PSD)	PW (PSD)	PD (PSD)	VT (PSD)	RR (PSD)	PS (PSD)
RS	-	0.98 (0.01)	0.03 (0.03)	0.006 (0.03)	−0.02 (0.03)	−0.04 (0.02)	0.03 (0.03)	0.04 (0.03)	−0.01 (0.02)	0.01 (0.01)	0.04 (0.02)
VS	0.99 (0.00)	-	0.03 (0.03)	0.01 (0.02)	0.02 (0.02)	−0.04 (0.03)	0.04 (0.03)	0.02 (0.03)	−0.01 (0.02)	0.01 (0.01)	0.03 (0.02)
ST	0.11 (0.21)	0.15 (0.22)	-	−0.01 (0.03)	0.01 (0.03)	FTC	0.001 (0.03)	0.001 (0.03)	0.03 (0.02)	0.01 (0.01)	0.03 (0.02)
LB	0.15 (0.29)	0.18 (0.31)	0.36 (0.30)	-	0.83 (0.01)	−0.06 (0.0.)	0.47 (0.02)	0.57 (0.02)	0.04 (0.02)	0.06 (0.01)	0.00 (0.02)
TB	−0.07 (0.22)	−0.06 (0.23)	0.21 (0.24)	0.97 (0.002)	-	0.33 (0.02)	0.36 (0.02)	0.56 (0.02)	FTC	0.01 (0.02)	0.01 (0.02)
SB	−0.24 (0.24)	−0.23 (0.26)	FTC	−0.64 (0.28)	0.79 (0.17)	-	−0.10 (0.02)	0.05 (0.03)	−0.01 (0.02)	0.01 (0.02)	0.00 (0.02)
PW	0.13 (0.26)	0.15 (0.25)	0.18 (0.27)	0.11 (0.39)	0.18 (0.27)	0.16 (0.33)	-	−0.24 (0.02)	FTC	0.04 (0.01)	−0.01 (0.02)
PD	0.03 (0.22)	0.09 (0.21)	0.19 (0.26)	0.74 (0.19)	0.69 (0.14)	0.49 (0.21)	−0.54 (0.20)	-	FTC	−0.02 (0.02)	0.02 (0.02)
VT	−0.02 (0.17)	−0.05 (0.18)	0.52 (0.26)	FTC	FTC	−0.06 (0.23)	FTC	FTC	-	-	-
RR	0.35 (0.20)	0.41 (0.23)	−0.08 (0.22)	−0.48 (0.44)	−0.62 (0.32)	−0.33 (0.29)	−0.22 (0.31)	−0.23 (0.35)	-	-	-
PS	0.33 (0.24)	0.36 (0.25)	−0.24 (0.39)	−0.47 (0.48)	−0.59 (0.37)	−0.24 (0.33)	−0.62 (0.24)	FTC	-	-	-

^a^ RS, responsiveness score; ST, shave time; VS, vocalization score; LB, total number of piglets born alive; TB, total number of piglets born; SB, total number of stillborn piglets; PW, number of piglets weaned; PD, number of piglet deaths; VT, vaginal temperature; RR, respiration rate; PS, panting score; FTC, failure to converge.

**FIGURE 2:**
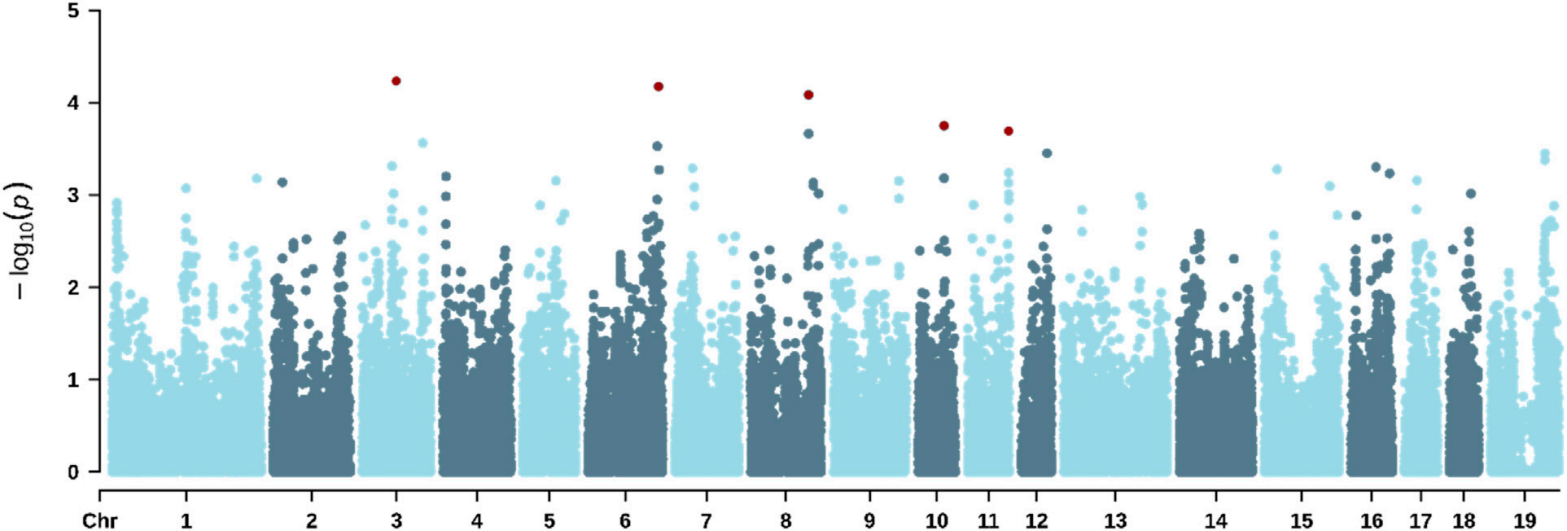
Manhattan plot for the responsiveness score during a standardized hair shaving procedure in lactating sows under heat-stress conditions. The red dots indicate significant SNPs.

**FIGURE 3:**
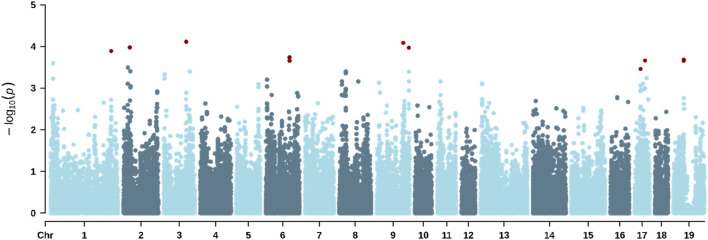
Manhattan plot for the shave time during a standardized hair shaving procedure in lactating sows under heat-stress conditions. The red dots indicate significant SNPs.

**FIGURE 4:**
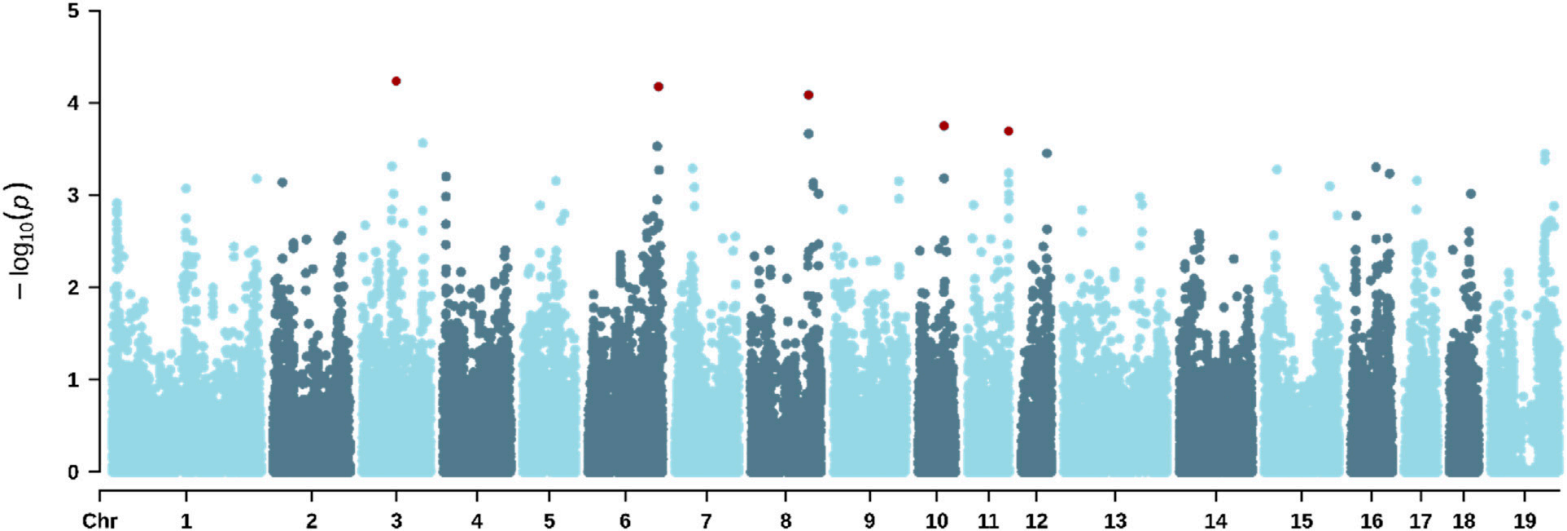
Manhattan plot for the vocalization score during a standardized hair shaving procedure in lactating sows under heat-stress conditions. The red dots indicate significant SNPs.

The GWAS enabled the identification of significant SNPs associated with all three traits studied. Table 8 shows the genomic location of all SNPs significantly associated with the traits studied. Figure 2 shows the Manhattan plot for RS, where six significant SNPs were identified on *Sus scrofa* chromosomes SSC3, SSC6, SSC8, SSC10, SSC11, and SSC12, with minor allele frequencies ranging from 0.045 to 0.408. Figure 3 shows the Manhattan plot for ST, highlighting 13 significant markers located on SSC1, SSC2, SSC3, SSC6, SSC9, SSC17, and SSC19. Figure 4 shows the Manhattan plot for VS, indicating six significant markers located on SSC3, SSC6, SSC8, SSC10, SSC11, and SSC12.

**TABLE 8:** Location of significant genomic markers organized based on trait, chromosome, and position.

Trait^a^	CHR^b^	Position (bp)	Allele frequency^c^	Variance explained
RS	SSC6	149,140,103	0.407	2.33 × 10^−7^
	SSC10	54,710,198	0.044	4.15 × 10^−8^
	SSC11	86,175,659	0.368	2.03 × 10^−7^
	SSC12	54,106,001	0.347	2.12 × 10^7^
	SSC3	71,648,171	0.294	1.58 × 10^−7^
	SSC8	122,112,654	0.094	8.60 × 10^−8^
ST	SSC2	29,071,216	0.771	2.70 × 10^6^
	SSC9	124,711,496	0.011	2.20 × 10^−7^
	SSC6	111,342,776	0.015	3.02 × 10^7^
	SSC17	48,503,856	0.518	4.14 × 10^−6^
	SSC3	104,827,510	0.362	3.45 × 10^−6^
	SSC17	27,499,717	0.754	2.87 × 10^−6^
	SSC3	104,841,993	0.362	3.47 × 10^−6^
	SSC9	151,005,996	0.207	2.38 × 10^−6^
	SSC9	124,635,111	0.011	2.20 × 10^−7^
	SSC6	110,866,392	0.014	2.94 × 10^7^
	SSC1	283,184,938	0.628	3.97 × 10^−6^
	SSC19	47,212,962	0.136	1.70 × 10^−6^
	SSC19	47,302,768	0.136	1.70 × 10^−6^
VS	SSC6	149,140,103	0.407	2.33 × 10^−7^
	SSC10	54,710,198	0.044	4.15 × 10^−8^
	SSC11	86,175,659	0.368	2.03 × 10^−7^
	SSC12	54,106,001	0.347	2.12 × 10^7^
	SSC3	71,648,171	0.294	1.58 × 10^−7^
	SSC8	122,112,654	0.094	8.60 × 10^−8^

^a^ RS: responsiveness score; ST: shave time; VS: vocalization score.

^b^ Chromosome.

^c^ Frequency of the marker as it occurred within the sampled population of sows.

Table 9 shows the positional candidate genes harboring the significant SNPs identified for each trait. Gene annotation revealed 25 genes located on SSC3, SSC6, SSC8, SSC10, and SSC12 for RS and VS, whereas 22 significant genes, located on SSC2, SSC6, SSC9, and SSC17, were identified for ST. For RS and VS, 16 associated regions were classified as protein-coding biotypes, 8 were long noncoding RNAs (lncRNA), and 1 was identified as small nuclear RNA (snRNA). Similarly, for ST, 16 regions were classified as protein-coding biotypes, 5 as lncRNA, and 1 as snRNA. The Gene Ontology (GO) analysis did not identify any terms significantly associated with the studied traits.

**TABLE 9:** Genes annotated organized based on trait, chromosome, and then position with ensemble identifiers.

Trait^a^	CHR^b^	Position (bp)	Gene^c^	Gene ID	Gene biotype
RS	SSC6	149,140,103	*ENSSSCG00000003815*, *ENSSSCG00000032215*, *ENSSSCG00000060767*, *ENSSSCG00000050660*, and *ENSSSCG00000051600*	*ALG6* *FOXD3*	Protein coding Protein coding IncRNA IncRNA IncRNA
	SSC10	54,710,198	*ENSSSCG00000040615*, *ENSSSCG00000032444*, and *ENSSSCG00000055626*	*MALRD1* *PLXDC2* *MALRD1*	Protein coding Protein coding Protein coding
	SSC12	54,106,001	*ENSSSCG00000017991*, *ENSSSCG00000017993*, *ENSSSCG00000061105*, *ENSSSCG00000061443*, and *ENSSSCG00000053450*	*PIK3R5* *NTN1*	Protein coding Protein coding IncRNA IncRNA IncRNA
	SSC3	71,648,171	*ENSSSCG00000008317*, *ENSSSCG00000058412*, *ENSSSCG00000008314*, *ENSSSCG00000008318*, *ENSSSCG00000008319*, *ENSSSCG00000023258*, *ENSSSCG00000008321*, *ENSSSCG00000020736*, and *ENSSSCG00000057090*	*TEX261* *ANKRD53* *ATP6V1B1* *VAX2* *CD207* *CLEC4F* *FIGLA* *ADD2*	Protein coding Protein coding Protein coding Protein coding Protein coding Protein coding Protein coding Protein coding IncRNA
	SSC8	122,112,654	*ENSSSCG00000022353*, *ENSSSCG00000009188*, and *ENSSSCG00000019720*	*RAP1GDS1* *STPG2* *U6*	Protein coding Protein coding snRNA
ST	SSC2	29,071,216	*ENSSSCG00000013320*, *ENSSSCG00000013321*, *ENSSSCG00000041609*,and *ENSSSCG00000054408*	*PAX6* *ELP4*	Protein coding Protein coding Protein coding IncRNA
	SSC9	124,635,111	*ENSSSCG00000015557* and *ENSSSCG00000015558*	*NMNAT2* *SMG7*	Protein coding Protein coding
		124,711,496	*ENSSSCG00000015557*, *ENSSSCG00000015558*, and *ENSSSCG00000015559*	*NMAT2* *SMG7* *NCF2*	Protein coding Protein coding Protein coding
	SSC6	111,342,776	*ENSSSCG00000035965*, *ENSSSCG00000046533*, *ENSSSCG00000061859*, *ENSSSCG00000053006*, and *ENSSSCG00000054474*	*AQP4*	Protein coding IncRNA IncRNA IncRNA IncRNA
		110,866,392	*ENSSSCG00000003718*, *ENSSSCG00000003716*, *ENSSSCG00000003717*, and *ENSSSCG00000045029*	*TAF4B* *SS18* *PSMA8* *U2*	Protein coding Protein coding Protein coding snRNA
	SSC17	48,503,856	*ENSSSCG00000022777*, *ENSSSCG00000023306*, and *ENSSSCG00000007439*	*SLC35C2* *ELMO2* *CDH22*	Protein coding Protein coding Protein coding
		27,499,717	*ENSSSCG00000007100*	*SLC24A3*	Protein coding
VS	SSC6	149,140,103	*ENSSSCG00000003815*, *ENSSSCG00000032215*, *ENSSSCG00000060767*, *ENSSSCG00000050660*, and *ENSSSCG00000051600*	*ALG6* *FOXD3*	Protein coding Protein coding IncRNA IncRNA IncRNA
	SSC10	54,710,198	*ENSSSCG00000040615*, *ENSSSCG00000032444*, and *ENSSSCG00000055626*	*MALRD1* *PLXDC2* *MALRD1*	Protein coding Protein coding Protein coding
	SSC12	54,106,001	*ENSSSCG00000017991*, *ENSSSCG00000017993*, *ENSSSCG00000061105*, *ENSSSCG00000061443*, and *ENSSSCG00000053450*	*PIK3R5* *NTN1*	Protein coding Protein coding IncRNA IncRNA IncRNA
	SSC3	71,648,171	*ENSSSCG00000008317*, *ENSSSCG00000058412*, *ENSSSCG00000008314*, *ENSSSCG00000008318*, *ENSSSCG00000008319*, *ENSSSCG00000023258*, *ENSSSCG00000008321*, *ENSSSCG00000020736*, and *ENSSSCG00000057090*	*TEX261* *ANKRD53* *ATP6V1B1* *VAX2* *CD207* *CLEC4F* *FIGLA* *ADD2*	Protein coding Protein coding Protein coding Protein coding Protein coding Protein coding Protein coding Protein coding IncRNA
	SSC8	122,112,654	*ENSSSCG00000022353*, *ENSSSCG00000009188*, and *ENSSSCG00000019720*	*RAP1GDS1* *STPG2* *U6*	Protein coding Protein coding snRNA

^a^ RS, responsiveness score; ST, shave time; VS, vocalization score.

^b^ Chromosome.

^c^ Genes were identified using ENSEMBL database nomenclature.

The QTL annotation and enrichment analysis identified 98 QTL associated with the traits RS and VS, with 52% of them classified under the “*Meat and Carcass*” category, 30% as “*Health*,*”* and 7% as “*Reproduction*.” The remaining 11% QTLs were classified as “*Exterior*,” “*Production*,” and “*Meat and Carcass eQTL*.” For ST, 119 QTLs were identified and enriched, with 62% classified as “*Meat and Carcass*,*”* 26% as “*Health*,” 8% as “*Exterior and Production*,” and 4% as “*Reproduction*.*”*

## 4 Discussion

Improving heat tolerance is a key breeding goal in commercial pig populations, which are increasingly vulnerable to productivity losses due to increasing environmental temperatures and greater heat stress sensitivity (McConn et al., 2021; 2022). Consequently, there is growing interest in identifying novel indicator traits that can contribute to accelerating genetic progress for climatic resilience without compromising animal performance. Animal behavior under heat-stress conditions has been phenotypically linked to heat tolerance (Bonneau et al., 2021; Pontiggia et al., 2024; Byrd et al., 2025). In this context, we developed a behavioral ethogram suitable for implementation in commercial farm settings to evaluate the genetic background of behavioral traits and their genetic relationships with physiological indicators of heat stress, such as VT. We assessed the behavioral response of lactating sows during a standardized hair shaving procedure by quantifying RS, VS, and ST. To our knowledge, this is the first study investigating the genetic background of behavioral responses to standardized human handling tests, combined with both manual and automated measurements of heat stress response in lactating sows.

Understanding the genetic background underlying these behavioral and heat stress response traits is critical for the development of effective strategies to enhance the adaptability of pigs to increasingly variable environmental conditions. All studied traits were heritable, with heritability estimates ranging from 0.05 to 0.17. Notably, behavioral traits exhibited the highest heritability estimates among all studied traits. Although, to the best of our knowledge, no previous studies have reported heritability estimates for RS, VS, or ST in lactating sows, similar heritability values were observed in piglets for other behavioral traits, such as activity scores and back-test responses, with heritability estimates ranging from 0.15 to 0.19 (Rohrer et al., 2013). Moreover, maternal traits in this study had low heritability estimates, ranging from 0.05 for LB to 0.12 for PD, and these values are consistent with those previously reported in the Landrace and Yorkshire breeds (Kramer et al., 2021; Song et al., 2010). The physiological indicators of heat stress were also found to be heritable, as previously reported by Freitas et al. (2023). These traits are essential for capturing the underlying physiological mechanisms involved in heat stress response and, due to their heritable nature, can be incorporated into genomic breeding programs aimed at improving heat tolerance in pigs, especially during the lactation stage.

Although all traits evaluated in this study are heritable, the generally low heritability estimates suggest that although genetic factors influence these traits, environmental conditions still play a substantial role in their phenotypic variability. Therefore, improving management, facilities, nutrition, and other nongenetic factors is also equally important to mitigate the impact of heat stress in swine production as nongenetic factors can directly influence how animals respond to heat stress on a day-to-day basis, regardless of the animal’s genetic predisposition (Wang et al., 2020). The posterior standard deviations of the estimates were relatively large, indicating limited precision in the estimates, likely due to the sample size or high trait complexity.

In addition to evaluating the genetic background of behavioral traits, we also assessed their genetic relationship with reproductive and maternal ability traits and other indicators of heat stress. The strong genetic correlation (0.99 ± 0.01) observed between RS and VS indicates that these traits essentially represent the same underlying genetic variability. This was expected as RS is defined as a combination of VS and the posture score, indicating that VS is inherently embedded within RS. Therefore, the inclusion of both traits in a selection index may be redundant as they do not provide independent sources of genetic information. ST showed a moderate genetic correlation with VT (0.52), which is considered a trait of direct physiological response to heat stress. This moderate correlation may indicate that animals exposed to heat stress tend to be more agitated, as reflected in longer ST. Although studies directly evaluating correlations between VT and ST are limited, several reports have shown correlations between behavioral responses and physiological indicators of heat stress in pigs (Bonneau et al., 2021; Poullet et al., 2025). These findings support the results observed in the present study and suggest that behavioral traits may be genetically linked to a physiological indicator of heat stress. However, these estimates are hypothesis-generating and should be further validated in controlled experimental studies.

The genetic correlations with maternal traits were mainly low and nonsignificant, potentially highlighting the need for more complete records regarding the cause of death and cross-fostering events. The highest correlation between behavioral and maternal traits was between ST and LB, with an estimate of 0.36. This preliminary observation may arise from shared biological pathways between certain behavioral and maternal traits or from the pleiotropic nature of the genes. However, further studies are needed to fully understand the biological mechanisms underlying the behavioral response to hair shaving under heat stress and maternal traits. Maternal traits also showed moderate and negative genetic correlations with physiological indicators of heat stress response, which, despite the relatively high standard errors, may still be biologically relevant. For instance, RR and TB showed a correlation of −0.62 (±0.32), whereas RR and LB showed a correlation of −0.48 (±0.44). Given that RR is an indicator of heat stress response, its negative correlation with TB and LB suggests that higher temperatures may result in smaller litters and reduced numbers of piglets born alive. Previous studies have reported similar patterns, indicating that sows exposed to heat stress around farrowing can experience a reduction of approximately 0.015 live born piglets per 1 °C increase in ambient temperature (Wegner et al., 2016). These findings suggest that the negative correlations between maternal traits and heat stress indicators may have direct impacts on breeding programs. Therefore, resilience-related traits should be considered in selection indices to mitigate the risk of reproductive losses under challenging thermal conditions.

Cross-fostering was not considered in this study because information on this management practice was unavailable. However, cross-fostering can substantially affect the apparent maternal performance of individual sows as the birth sow provides both the direct maternal genetics and the prepartum environment. In contrast, the foster sow contributes to the postpartum maternal environment (Alves et al., 2018). In addition, cross-fostering can alter sow–piglet interactions and impact piglet growth (Robert and Martineau, 2001), and it may help explain the weak or inconsistent genetic correlations observed between behavioral and maternal ability traits. Therefore, these results should be interpreted with caution.

Genetic correlations between behavioral traits and heat stress indicators were low to moderate, with values ranging from 0.33 (RS × PS) to 0.52 (ST × VT), suggesting a shared genetic basis influencing both trait groups. These results suggest that genetic changes in one trait are expected to moderately impact the other traits at the genetic level. The positive and moderate genetic correlations observed between behavioral and physiological indicators of heat stress imply that pigs genetically predisposed to exhibit higher body temperatures also tend to display behaviors involving increased activity during shaving. This association may reflect an increased sensitivity to heat stress, which manifests through physiological and behavioral responses.

Most of the genetic correlations reported in the study showed large posterior standard deviations, which is due to the limited number of records included in the analyses. In future studies, collecting phenotypic records across multiple generations could improve the accuracy of genetic parameter estimates. Despite the low-to-moderate genetic correlations between sow behavior during hair shaving and physiological indicators of heat stress, RS (or VS) and ST could be valuable additional traits to include in a heat stress selection sub-index as behavioral assessments based on standardized protocols involving human observation over a fixed timeline are generally easier and more cost-effective to implement than physiological measurements. Additional behavioral tests that may better capture behaviors more related to heat stress response should also be investigated.

The GWAS enabled the identification of important genomic regions associated with variability in RS, VS, and ST. Notably, the same genomic regions and genes were identified for RS and VS, which was expected due to the high genetic correlation observed between these two traits. Several candidate genes potentially involved in the heat stress response were identified for RS and VS. The phosphoinositide-3-kinase regulatory subunit 5 (*PIK3R50*), located on SSC12, encodes a regulatory subunit of phosphoinositide 3-kinase gamma (PI3Kγ) and plays a critical role in cellular signaling pathways involved in inflammation and metabolism (Luo et al., 2024). Under heat-stress conditions, the PI3K-Akt signaling pathway may become particularly important due to its involvement in promoting cell survival and modulating immune responses. Heat stress can induce oxidative damage and apoptosis, and activation of the *PI3K* pathway can neutralize these effects by enhancing cellular resilience (Chen et al., 2024; Zhou et al., 2024). In pigs, the modulation of *PIK3R5* expression may, therefore, contribute to differential heat tolerance by influencing inflammatory signaling and protecting tissues from thermal damage. Another important gene associated with both RS and VS was Netrin-1 (*NTN1*) on SSC12, which is a multifunctional gene known for its role in axon guidance during neural development, but it also plays a significant role in inflammation, cell survival, and vascular homeostasis (Mentxaka et al., 2023). Under heat-stress conditions, tissue damage and inflammation are common, and *NTN1* is increasingly recognized for its anti-inflammatory properties, acting through multiple mechanisms to reduce immune cell recruitment and suppress pro-inflammatory cytokine expression, which can reduce inflammatory responses and promote tissue protection and repair (Mirakaj et al., 2011). In this context*, NTN1* may contribute to maintaining tissue integrity and reducing systemic inflammation, particularly in organs sensitive to thermal damage. In addition, Wang et al. (2019) reported that the *NTN1* gene plays an important role in the development of muscle and fat in pigs. As heat stress influences growth performance in livestock by altering gene expression in muscle and fat, the involvement of *NTN1* suggests a potential link between genetic regulation of tissue development and the physiological responses to thermal stress in pigs. Identifying these genes as significant for RS and VT reinforces the hypothesis that these behavioral traits are directly linked to physiological responses to heat stress in lactating sows.

A candidate gene identified for ST was neutrophil cytosolic factor 2 (*NCF2*), located on SSC9, which encodes a subunit of the NADPH oxidase complex and is critical to produce reactive oxygen species (ROS) during the innate immune response (Gauss et al., 2006; Tarazona-Santos et al., 2013). Heat stress leads to a significant increase in cellular oxidative stress by elevating the production of ROS (Habashy et al., 2019). The balance between ROS generation and the cell’s antioxidant defense mechanisms becomes critical for maintaining cellular health (Tchouagué et al., 2019). When heat stress overwhelms antioxidant defenses, oxidative damage to DNA, proteins, lipids, and other cellular components may occur (Akbarian et al., 2016). Although direct studies on *NCF2* in pigs are limited, neutrophils are known to play a critical antiviral role. Zhu et al. (2019) reported that during African swine fever virus infection, pigs exhibited neutrophilia (increased neutrophil counts). However, according to the authors, the recruitment of neutrophils to infection sites was suppressed due to the downregulation of key chemokines. This immune evasion strategy may compromise the effectiveness of neutrophil responses, potentially involving components such as *NCF2*. In this context, *NCF2* plays a key role in mediating oxidative bursts in neutrophils and other immune cells, which may be exacerbated during heat stress. In pigs, differential expression of *NCF2* under heat stress may reflect an adaptive immune response or contribute to oxidative tissue injury.

The identification of candidate genes such as *PIK3R5*, *NTN1*, and *NCF2*, which are associated with inflammatory response, immune regulation, and fat deposition, respectively, aligns with the observation that most QTLs identified for RS, VS, and ST in this study fall under the categories of “Health” and “Meat and Carcass.” For instance, heat stress in animals is known to reduce growth performance, feed intake, and body weight, likely due to impaired cellular energy production and increased oxidative stress (Wang et al., 2023). Another interesting finding is that for RS and VS, we identified QTLs associated with cortisol levels. Cortisol is a key hormonal indicator of stress and can increase rapidly under heat-stress conditions in both humans and other animals (Escribano et al., 2023; Follenius et al., 1982; Idris et al., 2024). Another important QTL associated with RS, VS, and ST was linked to oxygen saturation, and within the same region, the *NCF2* gene was also identified for the ST trait, supporting its potential role in oxidative stress and immune responses under heat stress. Heat stress is a significant environmental challenge that affects animals by leading to physiological and cellular disruptions. One critical consequence of heat stress is the disruption of oxygen utilization and induction of oxidative stress, which can impair cellular function and overall health (Wang et al., 2019; Wang et al., 2023). The identification of these QTLs suggests a potential genetic basis underlying the stress response mechanisms associated with heat-stress challenges.

Gene Ontology analysis is a crucial resource in genomics, enabling the elucidation and exploration of the functional roles of genetic elements and the understanding and interpretation of gene functions. In this study, no significant enrichment of biological processes was detected, suggesting that the genes may not act together in common pathways but rather reflect a broad spectrum of biological functions. The absence of significant GO enrichment may also be related to the polygenic nature of the studied traits, where many genes with small effects contribute to the phenotype. However, even though enriched processes were not identified, several genes were individually involved in key biological processes and pathways. These findings suggest that although enrichment processes were not observed, the individual genes may play complementary roles in regulating key biological mechanisms related to heat stress and behavioral traits in pigs.

The results obtained in this study are promising and contribute to understanding the behavioral variation among lactating sows exposed to heat stress. However, experimental validation is essential to confirm the biological roles of the identified candidate genes. Approaches such as gene expression assays of animals under heat-stress conditions and control groups could help determine whether these genes are differentially regulated in response to thermal challenges. Additionally, CRISPR-based functional studies can also provide a powerful strategy for directly understanding the contribution of individual genes to heat stress resilience in pigs.

## 5 Conclusion

In this study, we revealed significant behavioral variation among lactating sows exposed to heat-stress conditions during hair shaving, which can be effectively captured through a single manual observation by trained human observers. Despite the results obtained and the potential for implementation in breeding programs, automatic recording of such behaviors (e.g., camera-based assessments) may be more effective and provide greater precision. The behavioral traits assessed exhibited low-to-moderate heritability and showed little or no correlation (although some estimates had large posterior standard deviations) at the genetic level with some physiological indicators of heat stress response and maternal performance. Furthermore, genomic analyses identified significant associations among RS, VS, and ST and several genetic markers. Notably, some of these markers were located in proximity to genes previously implicated in thermotolerance, including *PIK3R5* and *NCF2*, underscoring the biological relevance of these behavioral phenotypes. Collectively, sow behavioral responses to a novel event under heat-stress conditions are heritable and likely highly polygenic but show little or no correlation with climatic resilience and maternal traits. Additional behavioral tests should be evaluated, and the genomic regions identified should be further validated in independent populations.

## Data Availability

The datasets presented in this article are not readily available because they are commercially sensitive. Requests to access the datasets for research purposes should be directed to the corresponding author (Dr. Luiz F. Brito; britol@purdue.edu).
