# Supplementary material for: Genetic background of behavior traits in lactating sows under heat-stress conditions and their relationship with heat tolerance and maternal performance traits
Source: Front Genet. 2025 Nov 17;16:1688262. doi: 10.3389/fgene.2025.1688262 (PMC12665351; doi:10.3389/fgene.2025.1688262)
Supplement: Supplementary file 1 [file DataSheet1.docx]

SUPPLEMENTARY MATERIAL

#
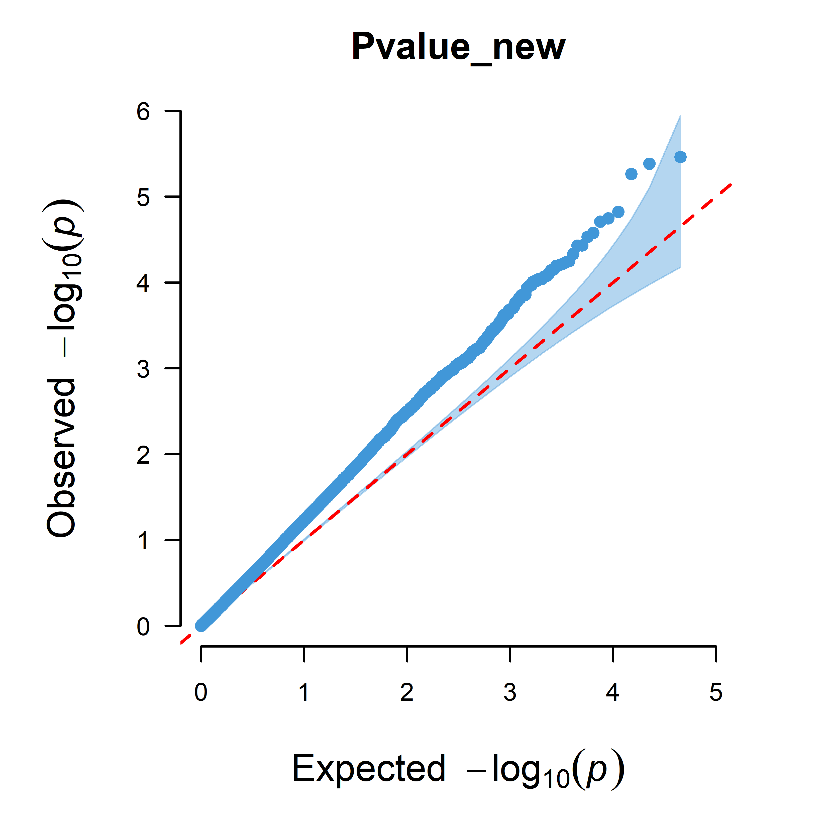

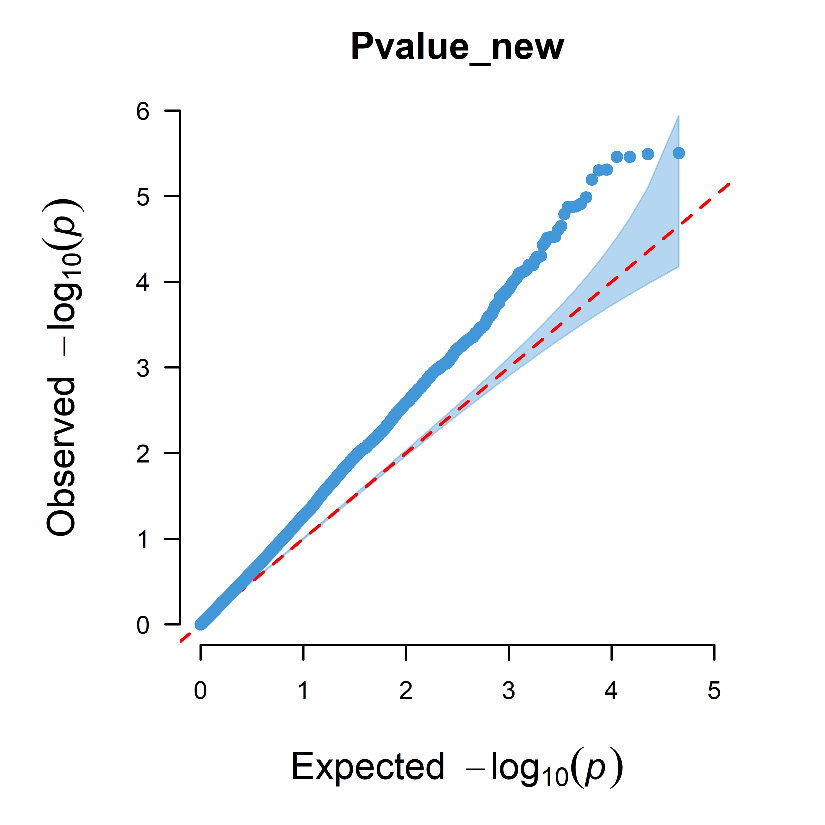


A

B


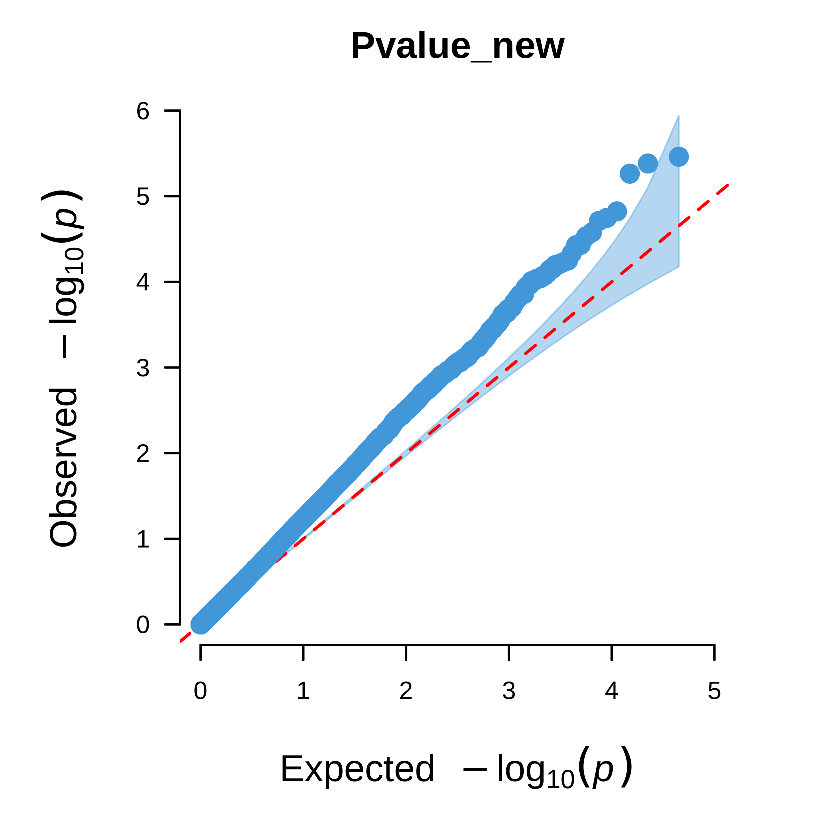


C

# Supplementary Material 1: Q–Q plots of adjusted p-values. A: Responsiveness score; B: Shave time; C: Vocalization score.
